# Risk Factors for Depression Among Civilians After the 9/11 World Trade Center Terrorist Attacks: A Systematic Review and Meta-Analysis

**DOI:** 10.1371/currents.dis.6a00b40c8ace0a6a0017361d7577c50a

**Published:** 2018-03-30

**Authors:** Abhinaba Chatterjee, Samprit Banerjee, Cheryl Stein, Min-Hyung Kim, Joseph DeFerio, Jyotishman Pathak

**Affiliations:** Department of Healthcare Policy and Research, Weill Cornell Medicine, New York, NY USA; Department of Healthcare Policy and Research, Weill Cornell Medicine, New York, NY USA; Department of Health and Mental Hygeine, Division of Epidemiology, World Trade Center Health Registry, New York, NY USA; Department of Healthcare Policy and Research, Weill Cornell Medicine, New York, NY USA; Department of Healthcare Policy and Research, Weill Cornell Medicine, New York, NY USA; Department of Healthcare Policy and Research, Weill Cornell Medicine, New York, NY USA

## Abstract

**Introduction:**

The development of depressive symptoms among the population of civilians who were not directly involved in recovery or rescue efforts following the 9/11 World Trade Center (WTC) terrorist attacks is not comprehensively understood. We performed a meta-analysis that examined the associations between multiple risk factors and depressive symptoms after the 9/11 WTC terrorist attacks in New York City among civilians including survivors, residents, and passersby.

**Methods:**

PubMed, Google Scholar, and the Cochrane Library were searched from September, 2001 through July, 2016. Reviewers identified eligible studies and synthesized odds ratios (ORs) using a random-effects model.

**Results:**

The meta-analysis included findings from 7 studies (29,930 total subjects). After adjusting for multiple comparisons, depressive symptoms were significantly associated with minority race/ethnicity (OR, 1.40; 99.5% Confidence Interval [CI], 1.04 to 1.88), lower income level (OR, 1.25; 99.5% CI, 1.09 to 1.43), post-9/11 social isolation (OR, 1.68; 99.5% CI, 1.13 to 2.49), post-9/11 change in employment (OR, 2.06; 99.5% CI, 1.30 to 3.26), not being married post-9/11 (OR, 1.59; 99.5% CI, 1.18 to 2.15), and knowing someone injured or killed (OR, 2.02; 99.5% CI, 1.42 to 2.89). Depressive symptoms were not significantly associated with greater age (OR, 0.86; 99.5% CI, 0.70 to 1.05), no college degree (OR, 1.32; 99.5% CI, 0.96 to 1.83), female sex (OR, 1.24; 99.5% CI, 0.98 to 1.59), or direct exposure to WTC related traumatic events (OR, 1.26; 99.5% CI, 0.69 to 2.30).

**Discussion:**

Findings from this study suggest that lack of post-disaster social capital was most strongly associated with depressive symptoms among the civilian population after the 9/11 WTC terrorist attacks, followed by bereavement and lower socioeconomic status. These risk factors should be identified among civilians in future disaster response efforts.

## Introduction

****The mental health consequences of the September 11, 2001 (9/11) World Trade Center (WTC) terrorist attacks in New York City (NYC) have been the focus of a substantial number of research endeavors over the past 15 years.^1^ A majority of this research has documented the etiology, prevalence, treatment, and risk factors for posttraumatic stress disorder (PTSD) in particular, as PTSD is reported to be the most common post-disaster associated condition.^2^ According to the Fifth Edition of the Diagnostic and Statistical Manual of Mental Disorders (DSM-5), PTSD is a conditional disorder that can develop among individuals after a qualifying trauma exposure.^3^ Qualifying trauma exposure must result from directly experiencing a traumatic event, being an eyewitness to trauma as it occurred to others, learning that a close associate suffered from a traumatic event, or experiencing repeated or extreme exposure to aversive details of a traumatic event.^3^

Probable depression is another commonly studied post-disaster mental health outcome. Probable depression refers to a positive screen on a depression symptom screening instrument, such as the Patient Health Questionnaire.^4^^,^^5^ PTSD and probable depression among trauma-exposed groups of the WTC terrorist attacks have been thoroughly described.^6^^,^^7^^,^^8^^,^^9^^,^^10^ Examples of frequently studied trauma exposed groups include firefighters, police, emergency medical technicians, first responders, and recovery and cleanup workers.

In contrast to trauma-exposed groups, the general civilian population of NYC, comprised of survivors, NYC residents, people working in the area, and passers-by on the day of the attacks, is an example of a mixed-exposure group. After the WTC terrorist attacks, members of the civilian population suffered from varying levels of exposure to trauma. Many of these exposures may have not met DSM-5 trauma exposure criteria.^11^^,^^12^ The general civilian population is not always included in studies of prevalence estimates and risk factors for post-disaster PTSD and probable depression because disaster mental health research is typically interested in understanding mental disorders in relation to trauma exposure.^13^ In particular, comprehensive analyses of post-disaster probable depression among civilians are lacking in the literature.

The unpredictable nature of terrorist attacks have introduced new definitions of affected populations in disaster mental health research, as the purpose of terrorism is to invoke fear and anxiety among civilians in general.^14^ Moreover, probable depression is not dependent on qualifying trauma exposure, so the population susceptible to probable depression after a disaster such as a terrorist attack is larger than the population susceptible to PTSD.^5^ Carefully identifying risk factors for probable depression among the civilian population that were associated with the 9/11 WTC terrorist attacks may better inform future disaster preparation efforts.

Over the past 15 years, a number of studies have screened for probable depression among affected populations and stratified their analysis of trauma-exposed groups and civilians, or sampled cohorts of civilians specifically.^15^^,^^16^^,^^17^^,^^18^^,^^19^^,^^20^^,^^21^ The aim of this study was to synthesize the results from research on 9/11-affected survivors, residents, and passers-by, summarize the influence of probable depression among these cohorts, and evaluate the associations between probable depression and various risk factors.

## Methods

*Search Strategy*

We conducted a meta-analysis following the Preferred Reporting Items for Systematic Reviews and Meta-Analyses (PRISMA) Statement.^22^ PubMed, Google Scholar, and the Cochrane Library were searched without language restriction from September, 2001 through July, 2016. Search terms included “World Trade Center”, “WTC”, “World Trade Center Disaster”, “WTCD”, “September 11”, “9/11”, “Depression”, “Major Depression”, “Probable Depression”, and “Mental Health” in various relevant combinations. Both published and unpublished sources of data were considered. References of studies and review articles were also manually searched to yield additional studies not found through the original search.

*Study Inclusion/Exclusion Criteria*

Two reviewers (A.C., and S.B.) screened the search results for eligibility. Disagreements between reviewers regarding the inclusion or exclusion of a study were resolved by a third reviewer (J.P.). Studies were eligible based on the following inclusion criteria: 1) original research article; 2) focused on the effects of the WTC terrorist attacks; 3) focused on adult populations based on age at the time of interview; 4) focused on civilians or reported separate analysis of civilians; 5) screened for depression using validated diagnostic criteria; 6) documented probable depression prevalence (number of patients in the cohort screening positive for depressive symptoms) or odds ratio (OR) among civilians in response to pre-defined risk factors; and 7) conducted within the NYC metropolitan area.

*Data Extraction*

Reported risk factors for probable depression were extracted and compared across studies. Our main exposure variables consisted of only risk factors that were similarly defined among at least two studies included in the meta-analysis. Probable depression prevalence, ORs, and standard errors corresponding to each risk factor were then extracted from each study. Our primary outcome was the OR for the association between the development of depression symptomatology and each risk factor. All ordinal and categorical risk factor variables were dichotomized to simplify outcome synthesis; the Cochran-Mantel-Haenszel method was used to evaluate ORs based on probable depression prevalence among combined groups and Woolf’s method was used to combine weighted averages of log ORs.^23^^,^^24^ Other data elements that were extracted from each article were author surname, publication year, inclusion criteria, method and time period of cohort recruitment, sample size, baseline demographic characteristics of the sample, and overall prevalence of depressive symptoms.

*Statistical Analysis*

We used the DerSimonian and Laird (DL) random-effects model^25^ to calculate pooled ORs and corresponding standard errors for each included risk factor. These pooled estimates were interpreted as summary effect sizes that expressed the common odds of screening positive for probable depression with the presence of a risk factor, versus absence of a risk factor. Weights were calculated by the inverse variance method.

Heterogeneity across studies was investigated by the Cochran Q test and measured using I² and H² statistics. We interpreted I² values of 0-25%, 26-50%, 51-75%, and 76-100% as unimportant, moderate, substantial, and considerable heterogeneity, respectively.^26^ Publication bias was evaluated visually by examining symmetry in funnel plots. Egger regression^27^ and the Begg-Mazumdar test^28^ were used to quantify asymmetry by providing an estimate of correlations between effect sizes and corresponding variances. All analyses were performed using R software version 3.2.3 with the metafor package.^29^

We conducted sensitivity analysis by stratifying studies by the type of diagnostic criteria used and the recruitment period midpoint. This allowed us to evaluate whether structured diagnostic interviews and symptom screening instruments yielded different findings, and whether more recent event exposure had a stronger influence on depressive symptoms. Prevalence estimates were weighted for the sample size of each study within a particular strata and averaged. Variance weighted least squares regression was used to test for temporal trend. Sensitivity analysis was also conducted by assessing how responsive our summary measures were to individual data. We repeatedly fit the DL model for each risk factor while censoring one study at a time.^30^

To account for multiple tests, corresponding to the number of risk factors identified, we applied the Bonferroni correction^31^ to adjust the width of our confidence intervals (CIs) while testing for significance. Associations were considered significant at an α = 0.05/10 = 0.005 level.

## Results

**** *Electronic Search and Selection of Studies*

We screened 2,103 search results and excluded 2,058 articles on the basis of their titles or abstracts for not being WTC related, not being an original research article, or not investigating depression symptomatology (Figure 1). We then identified 5 additional studies after screening references of reviews. Of the remaining 50 studies, 43 were excluded for using self-reported prior diagnosis or unclear screening measures of depression (n = 7), not reporting sufficient data that could be pooled for analysis (n = 11), focusing solely on responders or not separating civilians in their analysis (n = 19), using a sample that overlapped with another study and reported the same risk factors (n = 5), or not being conducted within the NYC metro area (n = 1). The remaining seven studies met the inclusion criteria for our meta-analysis.

**PRISMA Flow Diagram of Study Selection:**
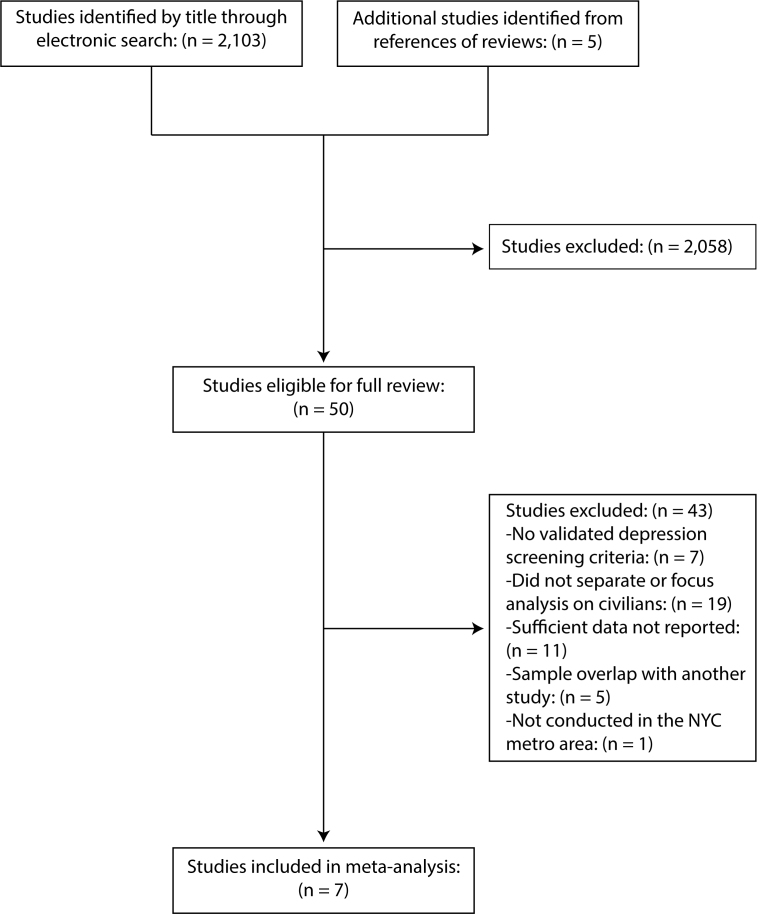

**** *Study Characteristics*

The 7 studies included were prospective cohort studies of civilians with sample sizes ranging from n = 149 to n = 22,026 (Table 1). Each study cohort was recruited independently from the civilian population, making the possibility of subjects participating in multiple studies, or overlap in the samples, unlikely. Study cohorts were also recruited at different times, and the mid-point of their recruitment periods were on average 2.94 years post 9/11.

**Figure:**
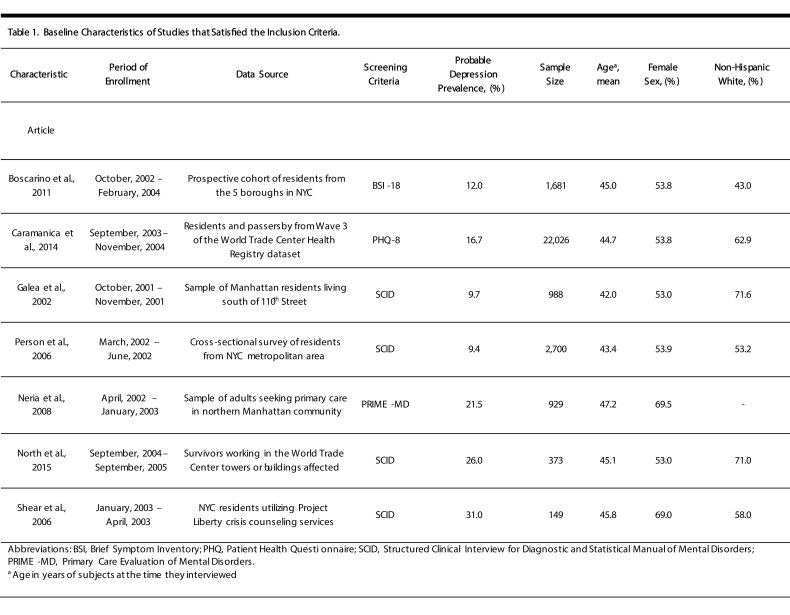
**Table 1:** Baseline Characteristics of Studies that Satisfied the Inclusion Criteria

Across studies, the average age of subjects at the time they were interviewed was 44.5 years, 61% were non-Hispanic white, and 54% were female. Probable depression prevalence ranged from 9.4% to 31.0%, and the overall prevalence was 15.9%. Of the 7 studies, 4 used a full Structured Clinical Interview for DSM-IV (SCID), which is considered the gold standard for diagnosing Major Depressive Disorder.^32^ The remaining studies used screening instruments to identify depression symptomatology, which is why we refer to our outcome as “probable depression” rather than as “clinically diagnosed depression”. The three studies reporting probable depression all used different screening instruments. Results from Caramanica et al. were adapted using Wave 3 of the WTC Health Registry,^16^^,^^20^ which used the 8-item Patient Health Questionnaire (PHQ-8), applying a cutoff at scores ≥ 10. This criteria has been shown to have sensitivity = 0.99 and specificity = 0.92 relative to SCID.^33^ Boscarino et al.^17^ used the Brief Symptom Inventory-18 (BSI-18), which has been shown to have sensitivity = 0.71 and specificity = 0.87,^34^ and Neria et al.^18^ used the Primary Care Evaluation of Mental Disorders (PRIME MD) Questionnaire, which has been shown to have sensitivity = 0.85 and specificity = 0.75,^35^ all relative to structured clinical interviews.

*Identification of Risk Factors*

There were 10 risk factors that were reported in more than one study. These 10 risk factors were divided into 3 categories: baseline demographic characteristics, post-disaster attributes, and exposure characteristics. Demographic characteristics included greater age, female sex, minority race/ethnicity (non-white or Hispanic/Latino ethnicity), no college degree, and lower income level. Post-disaster attributes included post-9/11 social isolation, post-9/11 change in employment status, and not being married post-9/11 at the time of interview. Exposure characteristics included knowing someone injured or killed during the attacks and direct exposure to 9/11 traumatic events.

Not all risk factors were defined identically in each study. No college degree, minority race/ethnicity, post 9/11 social isolation, not being married post-9/11, and direct exposure were reported both as multilevel categorical variables and as dichotomous variables comparing the presence of a risk factor to its absence. To facilitate pooling together these results, multi-level variables were dichotomized.

*Association between Probable Depression and Risk Factors*

Among the baseline demographic risk factors, only minority race/ethnicity (OR, 1.40; 99.5% CI, 1.04 to 1.88) and lower income level (OR, 1.25; 99.5% CI, 1.09 to 1.43) were significantly associated with elevated odds of probable depression (Figure 2). The pooled ORs for greater age (OR, 0.86; 99.5% CI, 0.70 to 1.05), female sex (OR, 1.24; 99.5% CI, 0.98 to 1.59), and no college degree (OR, 1.32; 99.5% CI, 0.96 to 1.83) indicated that these risk factors were not significantly associated with odds of probable depression.

**Forest Plot of Odds Ratios Stratified by Risk Factor.**
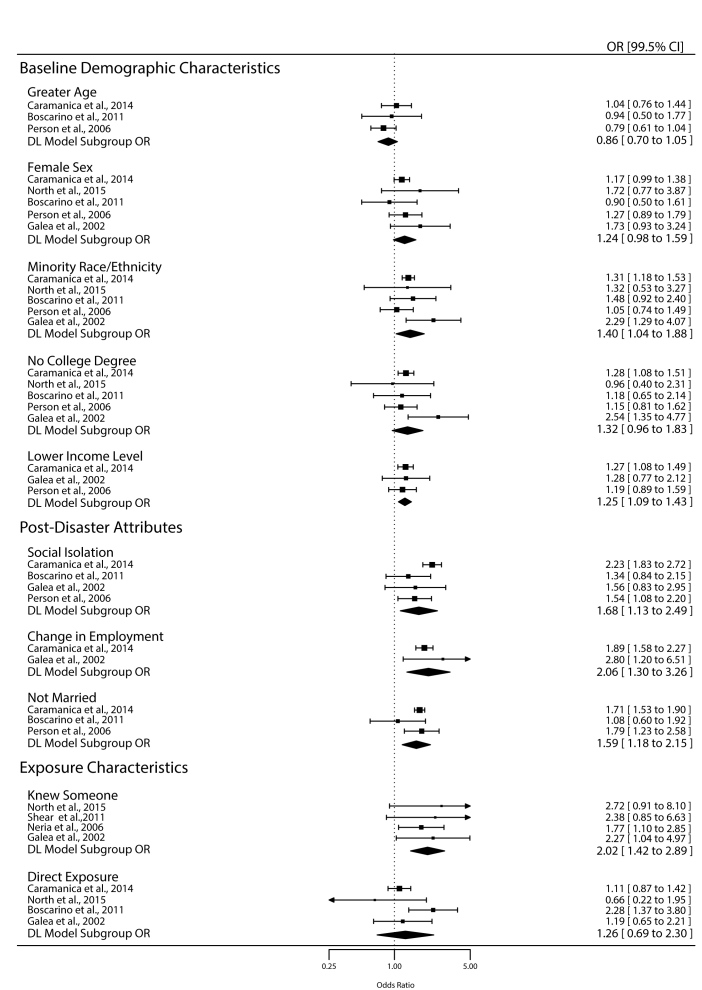

All three post-disaster attributes (social isolation, change in employment, and not being married) were significantly associated with elevated odds of probable depression, and their pooled ORs were among the largest summary effect sizes of the ten evaluated. The largest effect size in this category was the pooled OR for post-9/11 change in employment status (OR, 2.06; 99.5% CI, 1.30 to 3.26). The second largest effect size in this category was the pooled OR for social isolation (OR, 1.68; 99.5% CI, 1.13 to 2.49), and the smallest effect size was the pooled OR for not being married post-9/11 (OR, 1.59; 99.5% CI, 1.18 to 2.15).

Knowing someone injured or killed by the attacks and direct exposure to traumatic events were classified as exposure characteristics. The former significantly increased odds of probable depression (OR, 2.02; 99.5% CI, 1.42 to 2.89) while the latter was not significantly associated with probable depression (OR, 1.26; 99.5% CI, 0.69 to 2.30).

*Sensitivity Analysis for Diagnostic Criteria, Time from Disaster, and Removal of Individual Data*

Out of the 7 studies included, 4 used full structured diagnostic interviews and 3 used symptom screening instruments. Studies using symptom screeners detected higher rates of probable depression prevalence (Table 2). All associations for risk factors that were statistically significant in the primary analysis remained statistically significant in both sub-groups of diagnostic criteria. Additionally, while the magnitude of the estimates remained comparable, in the sensitivity analysis female sex was a significant risk factor based on structured interviews, and lack of college degree and direct exposure were significant risk factors among studies that used symptom screeners.

**Figure:**
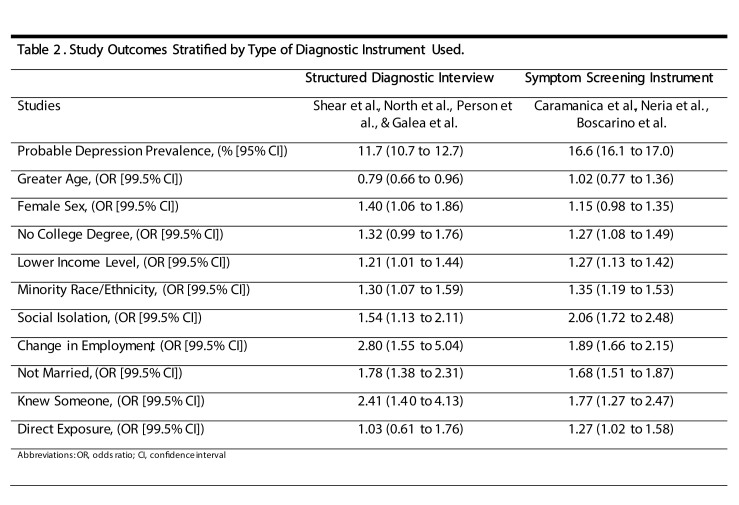
**Table 2:** Study Outcomes Stratified by Type of Diagnostic Instruments Used

We evaluated the prevalence of probable depression among all civilians included in studies that conducted interviews (midpoint of interview period) within a particular year (Supplementary Table). Although there were few studies included for each year, there was no significant temporal trend in depression prevalence (*P* = 0.25). We also assessed how stable our results were by evaluating the DL model for each risk factor after removing one study at a time (Table 3). This was only conducted for risk factors where results from more than two studies were synthesized. The female sex, minority race/ethnicity, no college degree, lower income level, and not being married post 9/11 risk factors lost or gained significance after removal of an individual study, however, all point estimates remained within the 95% confidence intervals of the original pooled estimates.

**Figure:**
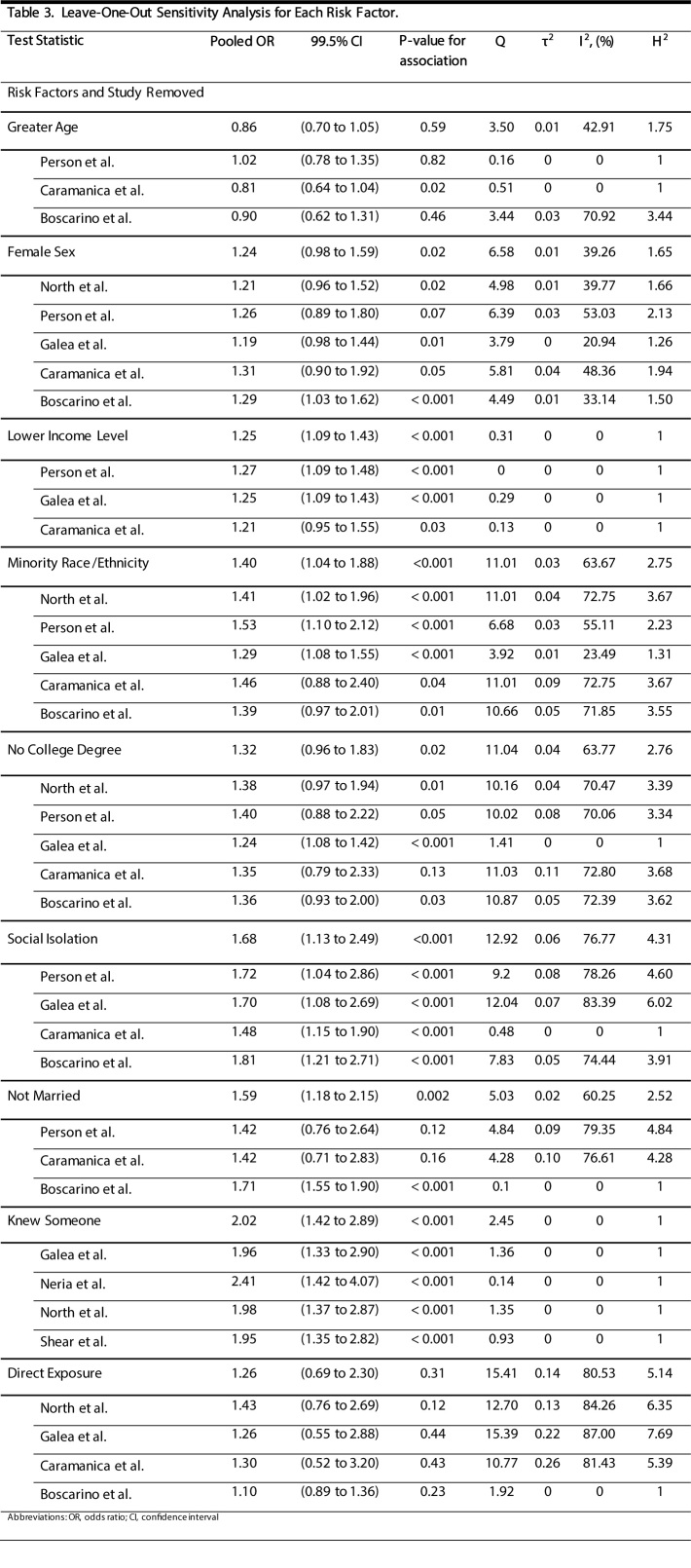
**Table 3:** Leave-One-Out Sensitivity Analysis for Each Risk Factor

*Publication Bias*

Visual inspection of funnel plots for each risk factor and for all studies combined did not reveal any notable publication bias (Supplementary Figures 1 & 2). Additionally, neither the Begg-Mazumdar rank correlation test (Kendall’s τ = 0.19, P = 0.10) nor the Egger regression test (z = 1.06, P = 0.29) detected statistically significant publication bias, although it is possible that the small number of included studies limits the power of this test.

## Discussion

This study draws attention to probable depression after the 9/11 WTC terrorist attacks among civilians in the NYC metro area. We meta-analyzed effect sizes for various factors associated with probable depression from studies conducted over the past 15 years. Our analysis identified baseline demographic characteristics, exposure types to the WTC terrorist attacks, and post-disaster attributes, and quantified their association with post-9/11 probable depression.

The prevalence of probable depression varied considerably across studies included in our meta-analysis. This can partly be attributed to differences in the characteristics of each cohort and the type of diagnostic criteria employed. We observed disparities between results yielded by symptom screening instruments and full structured diagnostic interviews that could have implications for future studies of post-disaster probable depression. Symptom screening instruments, which generally have high sensitivity for diagnosis of major depression,^33^^,^^34^^,^^35^ detected higher rates of overall probable depression prevalence (16.6%) compared to full structured diagnostic interviews (11.7%). Moreover, although all risk factors that were significantly associated with probable depression in the primary analysis remained significant in the sensitivity analysis, the associations for some risk factors, including female sex, no college degree, and direct exposure, were not consistent across diagnostic criteria. The magnitude of these differences were small, however, and can partially be explained by high heterogeneity or lack of power since just two studies were present in subgroups that were inconsistent with the primary analysis.

Of the baseline demographic characteristics that we evaluated, only being of a minority race/ethnicity and earning lower income were significantly associated with elevated odds of probable depression. Since race/ethnicity and income are known to correlate with socioeconomic status,^36^ our results may indicate an important association between post-disaster socioeconomic status and elevated risk of probable depression after a terrorist attack among civilians. Minority race/ethnicity has also been reported to be a risk factor for probable PTSD and depression among responders.^6^^,^^7^^,^^8^ Taken together, lower socioeconomic status could be used to identify target populations of mental health interventions so that the effect of intervention is maximized.

We also evaluated the association between WTC terrorist attack-related experiences and probable depression by focusing on two types of exposures: direct exposure to trauma, or knowing someone killed or injured by the attacks. Suffering the latter did not require being physically present in the vicinity of the attacks while they occurred, however, it was still significantly associated with elevated odds of probable depression. This finding strengthens evidence that probable depression may not be geographically constrained to the area affected after a disaster,^13^ and that bereavement may play an important role in driving the development of probable depression associated with terrorist attacks.^18^ Furthermore, the finding that direct exposure to 9/11 trauma was not significantly associated with elevated odds of probable depression suggests that terrorist attacks that expose few civilians directly to trauma may still lead to meaningful community-wide depressive symptoms.

An important limitation surrounding our discussion of the association between trauma exposure and probable depression is the definition of traumatic exposure that each study used. The studies included in this meta-analysis mainly found that traumatic exposure was not significantly associated with elevated odds of probable depression, but studies that used lenient definitions of what constituted trauma may have underestimated this association. We found, however, that the North et al.^13^ study used the most stringent definition of exposure to trauma, using “careful categorization of 9/11 trauma exposures based on the DSM-IV-TR definition,” and still did not find a significant association between exposure to trauma and elevated odds of probable depression. As categorization of trauma became more careful and strict among the studies included in this meta-analysis, the effect size for its association with probable depression did not increase. Therefore, it is difficult to argue that inconsistent criteria for what constituted trauma in different studies explains the lack of association with elevated odds of probable depression observed.

We also classified social isolation, post-9/11 change in employment status, and not being married post-9/11 as post-disaster attributes and identified their association with probable depression. Social support is the perception that one belongs to a supportive social network and has access to a variety of social integration sources.^37^ In the studies we encountered, social support was reported as the perceived number of social integration sources and was variably categorized. Our results suggest that a lack of social support, or social isolation, may play an important role in the development of probable depression after a terrorist attack; civilians without social support suffered 68% increased odds of probable depression after the WTC terrorist attacks. Similar results were observed for post-9/11 change in employment status and not being married post-9/11 as risk factors. The magnitudes of the pooled ORs for these three risk factors were among the four largest of the ten summary effect sizes evaluated in our study. Social integration resources, employment, and marriage are pivotal forms of social capital that influence how psychological stress affects civilians.^38^ We found that access to these resources generally had a stronger association with probable depression than exposure or baseline demographics after the 9/11 WTC terrorist attacks. The causal direction of this association, however, remains uncertain as social capital and probable depression were both measured at the same time in the studies included.

These results must also be interpreted in the context of the study’s limitations. Primarily, we could not include pre-existing psychopathology as a risk factor due to large inconsistencies in how studies addressed it when reported. Pre-existing psychopathology has been strongly associated with post-disaster depression and other mental health outcomes.^5^^,^^30^ Its exclusion is not intended to undermine the need to consistently identify, screen, and triage patients with pre-existing mental disorders requiring treatment after a disaster such as a terrorist attack. Second, we could not consistently adjust for several confounding factors, such as prior trauma, when calculating ORs from reported data in the included studies. Some studies adjusted for covariates and provided adjusted ORs, but these adjustments may vary for the included studies. Third, there was between-study variability in the time of cohort recruitment and questionnaire administration. Although we evaluated the influence of more recent event exposure in a sensitivity analysis and could not discern a significant trend in depression prevalence among cohorts with less recent event exposure, there may have been unaccounted confounding with cohort characteristics and specific risk factors. Fourth, we observed high heterogeneity in specific subgroups when calculating pooled ORs. This may stem from individual variation in the effect of the risk factor, but it also may be caused by variation in the original classification of risk factors among different studies. Being able to differentiate between the influence of being a direct eyewitness to trauma and being physically harmed during the events would have been useful in this context. Differentiating between changes in employment status that were related to the WTC terrorist attacks, versus changes in employment status that occurred for other reasons, is another example of how having more specific classification of risk factors in different studies may have strengthened our analysis.

## Conclusions

This meta-analysis identified risk factors for probable depression associated with the WTC terrorist attacks among mixed-exposure groups from the civilian population in the NYC metro area that was not directly involved in rescue or recovery efforts. We found that social isolation, change in employment, and not being married after the WTC terrorist attacks were three of the four most strongly associated risk factors for probable depression. Furthermore, we found that direct exposure to WTC related traumatic events was not significantly associated with probable depression, while knowing someone injured or killed by the attacks was. Knowing someone injured or killed was the second most strongly associated risk factor for probable depression in this meta-analysis, suggesting that probable depression among civilians after a terrorist attack may primarily be bereavement driven. Finally, we identified minority race/ethnicity and lower income as the only baseline demographic characteristics that predicted elevated odds of probable depression. This association suggests that further efforts are necessary to understand and address the influence of socioeconomic status and probable depression among civilians after terrorist attacks.

Our analysis allows for a better understanding of the associations between probable depression and risk factors among civilians who were not involved in rescue or recovery efforts by providing quantitative estimates for each association. The strong association between lack of social capital and depressive symptoms suggests that monitoring employment status and the availability of support should be points of focus in future studies and intervention efforts. We further recommend that persons of lower socioeconomic status or difficulties coping with bereavement receive greater attention, irrespective of exposure to trauma, after terrorist attacks. These efforts could improve the efficiency at which high-risk persons from the civilian population are identified in future mental health interventions and disaster response efforts.

## Corresponding Author

Abhinaba Chatterjee

Department of Healthcare Policy and Research

Weill Cornell Medicine

402 E 67th St

New York NY, USA 10065

Email: ac3642@columbia.edu

## Funding

This work was supported in part by NIH R01 MH105384. The funders had no role in study design, data collection and analysis, decision to publish, or preparation of the manuscript.

## Competing Interests

The authors have declared that no competing interests exist.

## Data Availability

All relevant data are reported in the manuscript.

## Ethics Statement

This analysis used only de-identified, publicly available, and previously published data sources. The need for informed patient consent was waived by the institutional review board and ethical committee at Weill Cornell Medicine.

## Appendix

PRISMA Checklist

**Figure:**
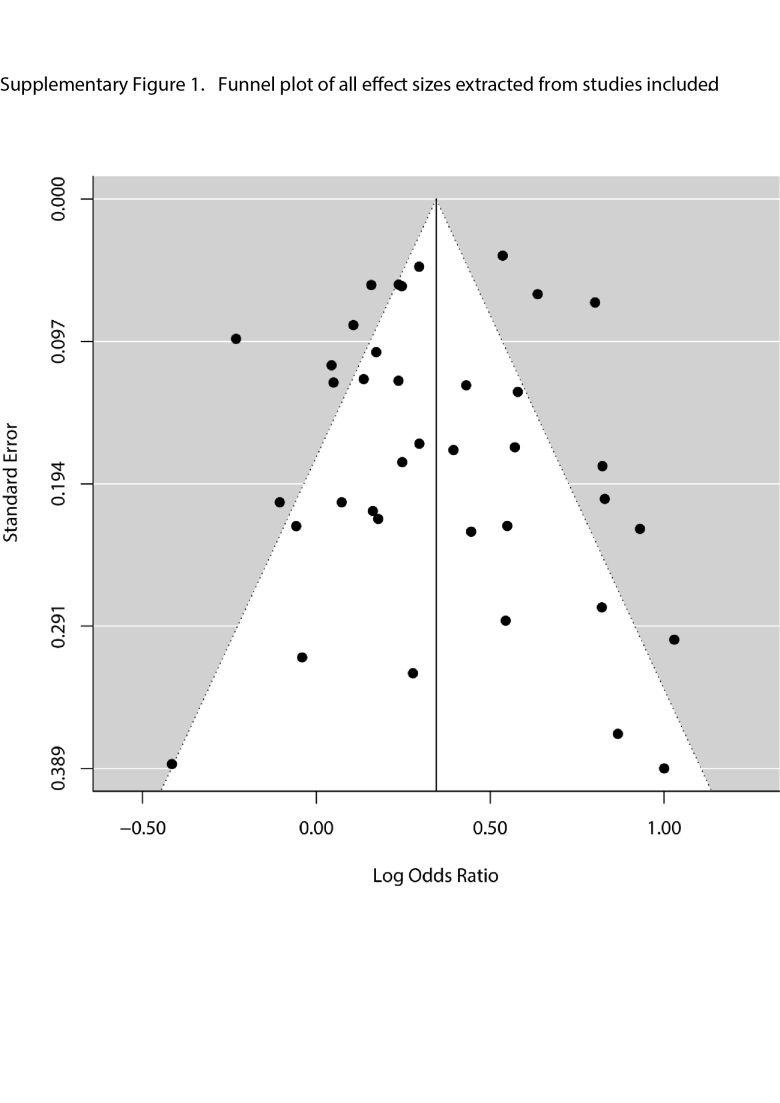
Supplemental Figure 1. Funnel plot of all effect sizes extracted from studies included.

**Figure:**
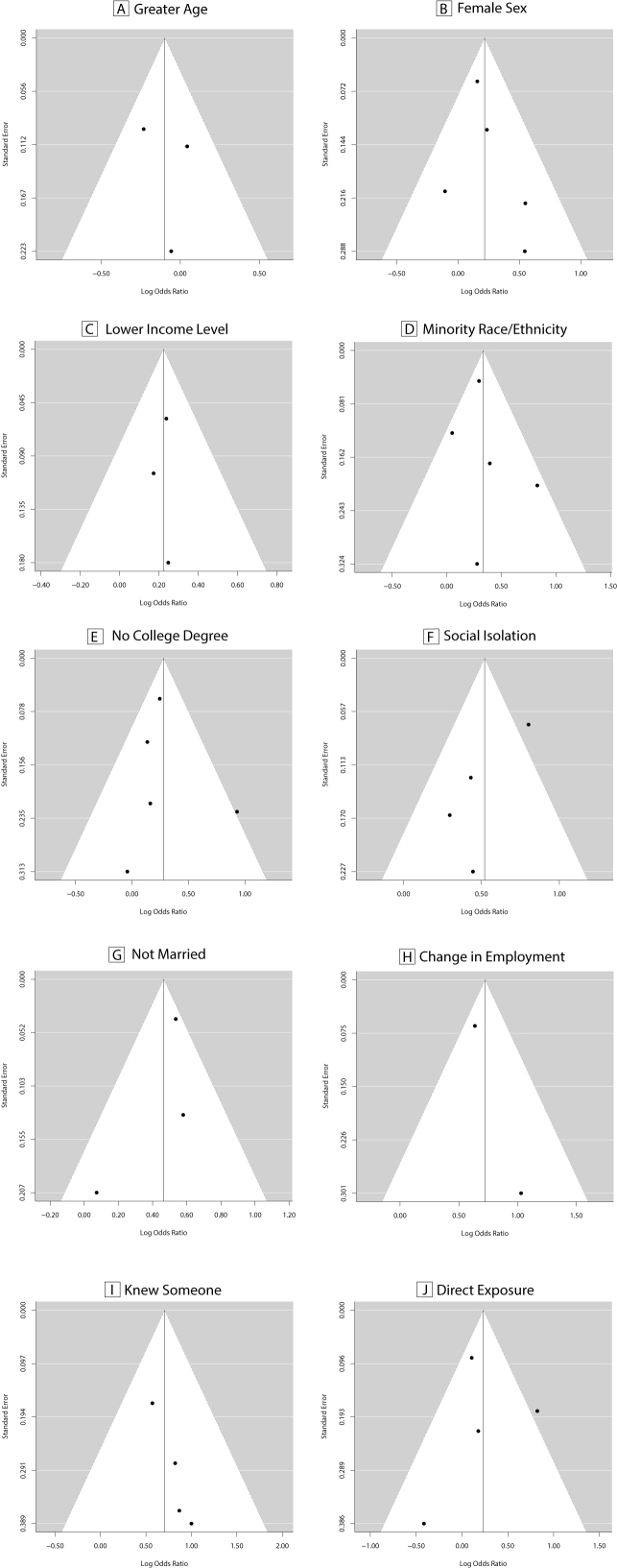
Supplemental Figure 2. Funnel plots of effect sizes for each risk factor.

**Figure:**
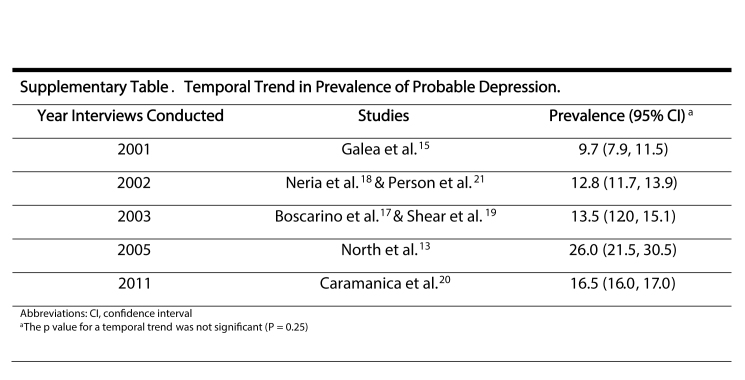
Supplemental Table. Temporal Trend in Prevalence of Probable Depression.
